# Efficacy of acupuncture for lifestyle risk factors for stroke: A systematic review

**DOI:** 10.1371/journal.pone.0206288

**Published:** 2018-10-26

**Authors:** David Sibbritt, Wenbo Peng, Romy Lauche, Caleb Ferguson, Jane Frawley, Jon Adams

**Affiliations:** 1 Australian Research Centre in Complementary and Integrative Medicine (ARCCIM), Faculty of Health, University of Technology Sydney, Sydney, New South Wales, Australia; 2 Nursing Research Centre, Western Sydney University & Western Sydney Local Health District, Blacktown Clinical & Research School, Blacktown Hospital, Sydney, New South Wales, Australia; Stanford University School of Medicine, UNITED STATES

## Abstract

**Background:**

Modifications to lifestyle risk factors for stroke may help prevent stroke events. This systematic review aimed to identify and summarise the evidence of acupuncture interventions for those people with lifestyle risk factors for stroke, including alcohol-dependence, smoking-dependence, hypertension, and obesity.

**Methods:**

MEDLINE, CINAHL/EBSCO, SCOPUS, and Cochrane Database were searched from January 1996 to December 2016. Only randomised controlled trials (RCTs) with empirical research findings were included. PRISMA guidelines were followed and risk of bias was assessed via the Cochrane Collaboration risk of bias assessment tool. The systematic review reported in this paper has been registered on the PROSPERO (#CRD42017060490).

**Results:**

A total of 59 RCTs (5,650 participants) examining the use of acupuncture in treating lifestyle risk factors for stroke met the inclusion criteria. The seven RCTs focusing on alcohol-dependence showed substantial heterogeneity regarding intervention details. No evidence from meta-analysis has been found regarding post-intervention or long-term effect on blood pressure control for acupuncture compared to sham intervention. Relative to sham acupuncture, individuals receiving auricular acupressure for smoking-dependence reported lower numbers of consumed cigarettes per day (two RCTs, mean difference (MD) = -2.75 cigarettes/day; 95% confidence interval (CI) = -5.33, -0.17; p = 0.04). Compared to sham acupuncture those receiving acupuncture for obesity reported lower waist circumference (five RCTs, MD = -2.79 cm; 95% CI: -4.13, -1.46; p<0.001). Overall, only few trials were considered of low risk of bias for smoking-dependence and obesity, and as such none of the significant effects in favour of acupuncture interventions were robust against potential selection, performance, and detection bias.

**Conclusions:**

This review found no convincing evidence for effects of acupuncture interventions for improving lifestyle risk factors for stroke.

## Introduction

Stroke is a major health issue with a significant burden upon quality of life and disability [1]. The control of stroke risk factors plays a vital role in reducing the risk of new or subsequent strokes of all types [2]. Three types of risk factors have been identified for stroke, including non-modifiable risk factors, medical risk factors, and lifestyle risk factors [2,3]. Lifestyle risk factors for stroke—hypertension, high cholesterol, smoking-dependence, alcohol-dependence, obesity, poor diet/physical inactivity—approximately accounted for 80% of the global risk of stroke [3]. Therefore, lifestyle risk factors for stroke are an ideal target for stroke prevention in comparison with other risk factors [4]. A growing stroke burden throughout the world suggests contemporary stroke prevention strategies for modifiable lifestyle risk factors may be insufficient and new effective approaches are needed [5]. However, the evidence for modification of lifestyle risk factors which are recommended by clinical guidelines for stroke management are not satisfactory [5,6].

Acupuncture is a traditional Chinese therapeutic intervention characterised by the insertion of fine metallic needles through the skin at specific sites (acupoints), with body and ears being the most common locations of acupoints [7]. Needles may be stimulated manually or by applying electric current [8]. There are various types of acupuncture treatments, such as needle acupuncture, electroacupuncture, acupressure, laser therapy, and transcutaneous electric acupoint stimulation (TEAS) [9]. Acupuncture has long been used for chronic diseases including musculoskeletal pain and hypertension [7]. The biological effects of acupuncture treatments, such as local inflammatory responses, anti-analgesia effects, and increase of opioid peptides, play an important role in the therapeutic effects of such therapy [10]. Nevertheless, the challenges inherent in designing and implementing rigorous acupuncture research may limit the understanding of the effectiveness of acupuncture, such as those relating to acupuncturists’ use of distinct syndrome classifications identified among people with the same condition and use of different skills when selecting and manipulating acupoints [11].

Using acupuncture to manage each lifestyle risk factor for stroke has attracted substantial and growing research interest over many decades. Previous reviews reported promising results of acupuncture use in controlling hypertension-associated symptoms [12], attaining weight loss [13], and reducing nicotine withdrawal symptoms [9]. In addition, WHO has indicated the effect of acupuncture for alcohol-dependence, in particular auricular acupuncture [14]. Nonetheless, a comprehensive systematic review assessing the effect of all forms of acupuncture for all identified lifestyle risk factors for stroke has not been conducted. As such, the aim of this paper is to identify and summarise the contemporary evidence of acupuncture interventions for lifestyle risk factors for stroke.

## Methods

The systematic review reported in this paper has been registered with PROSPERO (International prospective register of systematic reviews, #CRD42017060490).

### Search strategy

In accordance with the Preferred Reporting Items for Systematic Reviews and Meta-analyses (PRISMA) guideline, a systematic search of the literature was conducted using the MEDLINE, CINAHL/EBSCO, Scopus, and Cochrane Database of Systematic Reviews databases for studies published from January 1996 to December 2016. The lifestyle risk factors for stroke included in this systematic review are high blood pressure (hypertension & prehypertension), high cholesterol, obesity (overweight/obesity), smoking-dependence, alcohol-dependence, and physical inactivity. The literature search employed keyword and MeSH searches for terms relevant to ‘acupuncture’ and each lifestyle risk factor for stroke. Search terms used for each database are available in Table 1. Relevant randomised controlled trials (RCT) listed as references of published systematic review papers on selected lifestyle risk factors for stroke were also searched via Google Scholar by title, in order to include all relevant RCTs in this field.

**Table 1:** Search terms for the systematic review.

Acupuncture treatments	Acupuncture [MeSH Term & Keyword] OR Electroacupuncture [MeSH Term & Keyword] OR Electric stimulation [MeSH Term & Keyword] OR Acupressure [MeSH Term & Keyword] OR Laser acupuncture [MeSH Term & Keyword] OR *acupunctur*[Title/Abstract]	
AND		
Lifestyle stroke risk factors	High blood pressure	Hypertension [MeSH Term & Keyword] OR Blood pressure [MeSH Terms & Keyword] OR Hypertens* [Title/Abstract] OR Prehypertens* [Title/Abstract] OR Systolic [Title/Abstract] OR Diastolic [Title/Abstract] OR
	High cholesterol	Cholesterol [MeSH Term & Keyword] OR Triglycerides [MeSH Term & Keyword] OR Dyslipidemia [MeSH Term & Keyword] OR Epicholesterol [Title/Abstract] OR HDL [Title/Abstract] OR LDL [Title/Abstract] OR Triglyceride* [Title/Abstract] OR Hyperlipidem* [Title/Abstract] OR Lipidem* [Title/Abstract] OR
	Obesity	Obesity [MeSH Terms & Keyword] OR Overweight [MeSH Terms & Keyword] OR Metabolic syndrome [MeSH Terms & Keyword] OR Obes* [Title/Abstract] OR Adiposity [Title/Abstract] OR Adipos* [Title/Abstract]
	Alcohol-dependence/abuse	Alcohol [MeSH Terms & Keyword] OR Alcohol* [Title/Abstract]
	Smoking-dependence/abuse	Smoking [MeSH Terms & Keyword] OR Smok* [Title/Abstract]
	Physical inactivity	Exercise [MeSH Terms & Keyword] OR Exercis* [Title/Abstract]

* truncation symbol for literature search.

### Selection criteria

#### Types of studies

Studies were eligible for inclusion if they met the following criteria: (1) RCTs focusing on the efficacy and safety of acupuncture for lifestyle risk factors for stroke; (2) conducted in humans; (3) published in a peer-reviewed English language journal with abstracts; (4) reported primary data findings. Exclusion criteria were (1) RCT protocols or observation of a RCT of this research area; (2) quasi-/pseudo-RCTs and cross-over RCTs (3) studies focusing on the efficacy and safety of acupuncture treatment(s) for stroke or post-stroke symptoms; (4) studies focusing on the efficacy and safety of acupuncture treatment(s) for the complications of stroke risk factors; and (5) conference abstracts.

#### Types of interventions

There was no limitation on the forms of (traditional) acupuncture and the frequency and duration of the intervention. However, contemporary acupuncture such as trigger points and dry needling was not eligible for inclusion in this review.

#### Types of outcome measures

Only anthropometric parameters and the widely used indicators of each lifestyle risk factor for stroke were included. The primary outcomes were a change in systolic blood pressure (SBP) and/or diastolic blood pressure (DBP) for hypertension-focused RCTs; triglycerides, LDL/HDL cholesterol for hyperlipidemia/dyslipidemia-focused RCTs; body weight (BW), body mass index (BMI), waist circumference (WC) for obesity-focused RCTs; alcohol craving, completion rate of treatment, withdrawal symptoms for RCTs focusing on alcohol-dependence; withdrawal symptoms, daily cigarette consumption, abstinence rate for RCTs focusing on smoking-dependence; physical activity minutes/day and cardiorespiratory fitness for physical inactivity-focused RCTs.

### Data extraction

Title and abstracts of all citations identified in the search were imported to Endnote (Version X8) and duplicates removed. These citations were independently reviewed for eligibility by two authors (WP and RL) and the full texts of ambiguous articles were retrieved if consensus was not reached. Any disagreements were assessed by a third author. We contacted authors regarding raw data of their RCTs where necessary for meta-analysis. Where we failed to obtain such raw data, the RCT had to be excluded in the meta-analysis. According to the RCT description in the articles included, raw data were extracted from post-intervention effect and/or follow-up (long-term) effect.

Data were extracted into a pre-determined table (Table 2) and checked for coverage and accuracy by two authors independently. Table 2 includes detailed information on sample size, inclusion criteria, participants’ characteristics, intervention groups, add-on strategy, results of outcome measures, and side-effects. Both statistically significant within-group and/or between-group effect of acupuncture interventions for each lifestyle risk factor for stroke were recorded if reported.

**Table 2:** Summary of the included studies.

	Sample	Treatment intervention	Control intervention	Add-on strategy^a^	Results	Side-effects
**Alcohol-dependence**						
Rampes et al. 1997 UK[21]	59 randomized and 27, 26 completed at Wks 8, 24.	*1*. *Specific auricular electroacupuncture group* Acupoints: Lung, Shenmen, Sympathetic; *2*. *Nonspecific auricular electroacupuncture group* Acupoints: Elbow, Internal secretion; Manipulation: 100Hz frequency, 30 min/session, Weekly, 24 Wks	Individual alcohol counsellor/group therapy, 6 Wks	Conventional treatment	Alcohol craving: Significant within-group effect—TG1 & TG2 (8Wk)	Drowsiness; transient bleeding on needle removal; pain
	TG1: n = 23, 4 F, age 38y; TG2: n = 20, 3 F, age 40y; CG: n = 16, 6 F, age 42y;					
	*Inclusion*: alcohol withdrawal (DSM-III-R); 18–65 years; no previous acupuncture use					
Sapir-Weise et al. 1999 Sweden[20]	72 randomized and 72, 59, 51 completed at Wk 10, Mos 3, 6.	*Auricular acupuncture group* Acupoints: Lung, Shenmen, Sympathetic; Manipulation: nurses administered. 45 mins/session, Weekly (2-Wk), 3 times/Wk (4-Wk), twice/Wk (4-Wk)	*Non-specific acupuncture group* Acupoints: 3-5mm from the real acupoints; Same manipulation	Conventional treatment	Drinking days/alcohol craving: NS	N/A
	TG: n = 36, 11 F, age 47y; CG: n = 36, 10 F, age 45y;					
	*Inclusion*: alcohol withdrawal (DSM-III-R); no previous drug use					
Bullock et al. 2002 USA[22]	503 randomized and 356, 289, 247, 220 completed at Wk 3, Mos 3, 6, 12.	*1*. *Specific auricular acupuncture group* Acupoints: Liver, Lung, Shenmen, Sympathetic; *2*. *Nonspecific auricular acupuncture group* Acupoints: 5mm from the specific acupoints; 3. *Symptom-based auricular acupuncture group* Acupoints changed daily; Manipulation: acupuncturists administered. No manipulation. 40 mins/session, Weekly, 3 Wks	*Conventional treatment group* Detoxification, inpatient treatment, etc., 3 Wks	Conventional treatment	Alcohol withdrawal symptoms: Significant between-group effect—TG3>TG1 (12-Mo F/U)	N/A
	TG1: n = 132, 65 F, age 39y; TG2: n = 133, 66 F, age 38y; TG3: n = 104, 52 F, age 38y; CG: n = 134, 67 F, age 38 y;					
	*Inclusion*: alcohol≥3 days/Wk; 18–66 years; on-site≥14 days; blood platelet>22,000; no medications for alcohol abuse					
Karst et al. 2002 Germany[19]	34 completed.	*Auricular-body acupuncture group* Auricular acupoints: Kidney, Liver, Lung, Shenmen, Sympathetic; Body acupoints: DU20, Extra1, LI4; Manipulation: 30 mins/session, daily, 10 sessions	*Sham group* Same acupoints/manipulation; Needles without tips	Carbamazepine	Alcohol withdrawal symptoms: Significant within-group effect—TG	N/A
	TG: n = 17, 2 F, age 46y; CG: n = 17, 2 F, age 41y;					
	*Inclusion*: alcohol withdrawal (ICD-10); >18 years; no previous acupuncture use; no addiction to drugs					
Trumpler et al. 2003 Switzerland[18]	48 randomized and completed.	*1*. *Laser auricular acupuncture group 2*. *Needle auricular acupuncture group* Acupoints: prescribed individually; Manipulation: acupuncturists administered, daily, 6 days. 830nm infrared laser stimulation, 1 min per acupoint; no manipulation of the needle acupuncture, <40 mins/session	*Sham laser group* Same acupoints; No activated laser beam	Clomethiazole; benzodiazepines if necessary; maintain other drugs before study	Alcohol withdrawal symptoms: NS	Convulsion (TG)
	TG1: *n* = 17, 7 F, age 43y; TG2: *n* = 15, 5 F, age 45y; CG: *n* = 16, 8 F, age 49y;					
	*Inclusion*: alcohol withdrawal (DSM-IV); 18–65 years; no addiction to other drugs					
Kunz et al. 2007 Germany[17]	109 randomized and 74 completed.	*Auricular acupuncture group* Acupoints: Kidney, Liver, Lung, Shenmen, Sympathetic; Manipulation: psychiatrists/nurses administered. Needle stimulation, 45 mins/session, daily at 12:15PM, 5 days	*Aromatherapy group* 45 mins/session, daily at 12:15PM, 5 days	Carbamazepine or oxcarbazepine; benzodiazepines	Alcohol withdrawal symptoms/alcohol craving: NS	Pain, mild bleeding (TG); agitation, sneezing, sore throat (CG)
	TG: n = 55, 10 F, age 48y; CG: n = 54, 10 F, age 44y;					
	*Inclusion*: alcohol withdrawal (ICD-10); alcohol≥10 days; 18–65 years; no addiction to other drugs					
Lee et al. 2015 Korea[16]	20 randomized and completed.	*Body acupuncture group* Acupoint: KI9; Manipulation: oriental medical doctors administered. 15 mins/session, twice/Wk, 4 Wks	*Sham group* Same acupoints/manipulation; Needles without tips	N/A	Alcohol craving: NS	N/A
	TG: n = 10, age 43y; CG: n = 10, age 45y;					
	*Inclusion*: alcohol withdrawal (DSM-IV); male; no addiction to other drugs					
**Smoking-dependence**						
He et al. 1997 Norway[23,24]	46 randomized (age 39y) and 44, 38, 33 completed at Wk 3, Mo 8, Year 5.	① *Body electroacupuncture*, ② *auricular acupuncture*, ③ *auricular acupressure group* Acupoints: ① LU6, LU7; ② Lung, Mouth, Shenmen; ③ Endocrine, Hunger, Lung, Mouth, Shenmen, Trachea; Manipulation: acupuncturists administered, 3 Wks. ① 3Hz frequency, 20 mins, twice/Wk; ② needle stimulation, 20 mins, twice/Wk; ③ Vaccariae seeds acupressure 100 repeats/time, 4 times/day	*Non-specific acupuncture group* Acupoints: ① LI10, SJ8; ② Knees, Lumbar vertebra, Neck; ③ Buttock, Knees, Lumbar vertebra, Neck, Shoulder, Shoulder joint; Same manipulation	N/A	Daily cigarette consumption, desire to smoke: Significant within-group effect—TG (8-Mo/5-year F/U), CG (8-Mo F/U); Significant between-group effect—TG>CG (8-Mo F/U);	N/A
	TG: n = 26, 18 F, age 38y; CG: n = 20, 10 F, age 40y;				Cotinine concentrations: Significant within-group effect—TG;	
	*Inclusion*: smoking≥5 years and 10–30 cigarettes/day last year; heathy; no co-intervention for smoking				Smoking cessation rate: Significant between-group effect—TG>CG	
Waite & Clough 1998 UK[25]	79 randomized and 78 completed at Wk 2, Mos 2, 4, 6.	*Auricular electroacupuncture plus acupressure group* Acupoints: Lung; Manipulation: general practitioners administered, 2 Wks. 4Hz frequency, 20 mins/session; Chinese cow herb seed acupressure when feeling craving	*Sham group* Same acupoints/manipulation; Superficially placed needles	N/A	Smoking cessation rate: Significant between-group effect—TG>CG (6-Mo F/U)	Soreness, itch, pain of ears (TG); soreness, itch of ears (TG & CG)
	TG: n = 40, 18 F, age 24-67y; CG: n = 38, 16 F, age 23-69y;					
	*Inclusion*: ≥10 cigarettes/day; >18 years; no previous acupuncture use					
White et al. 1998 UK[26]	76 randomized and 52 completed at Wk 2, Mo 9.	*Auricular electroacupuncture group* Acupoints: Lung; Manipulation: acupuncturists administered. 100Hz frequency increase to above the threshold of sensation, 20 mins/session, 2 Wks	*Sham group* Superficially placed needles on location not acupoints	N/A	Smoking cessation rate: NS	N/A
	TG: n = 38, 21 F, age 41y; CG: n = 38, 18 F, age 43y;					
	*Inclusion*: ≥15 cigarettes/day; >21 years; no previous acupuncture use					
Georgiou et al. 1998 UK[27]	265 randomized and 216, 175, 63 completed at Wk 1, Mos 1, 3 (age 43y).	*Auricular electroacupuncture group* Acupoints: SJ17, SJ18; Manipulation: maximum 1-hour stimulation either 10Hz continuous frequency or 7-14Hz modulated frequency, 1 Wk	*Non-specific acupuncture group* Acupoints: SI15; Manipulation: stimulation machines disconnected from the electrodes	N/A	Smoking cessation rate/withdrawal symptoms/craving: NS	N/A
	TG: n = 108; CG: n = 108;					
	*Inclusion*: >10 cigarettes/day last year; >18 years; no co-intervention for smoking					
Cai et al. 2000 Singapore[29]	330 randomized and 268 (68 F), 208 completed at 6-session, Mo 3.	*Laser auricular acupuncture group* Acupoints: Lung, Mouth, Shenmen, Sympathetic; Manipulation: 6328A wavelength, 1mm diameter, 4 mins/session, 6 sessions	*Sham group* Same acupoints/manipulation; No laser ray	N/A	Daily cigarette consumption/Smoking cessation rate: NS	Headache, giddy, nausea, vomiting (TG: 20; CG: 21)
	TG: n = 128; CG: n = 140;					
	*Inclusion*: smoking≥3 Mos and ≥5 cigarettes/day; 12–18 years					
Bier et al. 2002 USA[31]	141 randomized (71 F, age 46y) and 108, 48 completed at Mos 1, 18.	*1*. *Auricular-body acupuncture plus education group 2*. *Auricular-body acupuncture group* Auricular acupoints: Kidney, Liver, Lung, Shenmen, Sympathetic; Body acupoints: LI4; Manipulation: acupuncturists administered. No needle stimulation. 30 mins/session, 4 Wks;*Educational program*: behavioral training, social support, relapse prevention techniques, 5 Wks	*Sham acupuncture plus education group* Acupoints: 5mm from the real acupoints; Same manipulation/education	N/A	Daily cigarette consumption, Smoking cessation rate: Significant between-group effect—TG1>CG>TG2 (after treatment)	Minor bleeding on needle removal (both TGs)
	TG1: n = 45; TG2: n = 38; CG: n = 58;					
	*Inclusion*: quitting smoking without success≥1; >18 years; no addiction to other drugs					
White et al. 2007 UK[28]	24 randomized and 19, 7 completed at Wks 1, 6.	*Auricular acupressure group 1* Acupoints: Lung, Shenmen; *Auricular acupressure group 2* Acupoints: Lung; Manipulation: researchers administered. Beads pressed when feeling craving, 6 Wks	*No intervention group*	NRT, group behavioral therapy	Withdrawal symptom: NS	N/A
	TG1: n = 6, 2 F, age 51y; TG2: n = 6, 5 F, age 40y; CG: n = 7, 7 F, age 44y;					
	*Inclusion*: ≥10 cigarettes/day; >18 years; no co-intervention for smoking					
Wu et al. 2007 Taiwan[33]	118 randomized and completed at Wk 8, Mo 6.	*Auricular acupuncture group* Acupoints: Lung, Mouth, Shenmen, Sympathetic; Manipulation: acupuncturists administered. 8 Wks	*Non-specific acupuncture group* Acupoints: Elbow, Eye, Knee, Shoulder; Same manipulation	N/A	Withdrawal symptom: Significant within-group effect—TG (after treatment);	Tenderness sensation (n = 50), dizziness (n = 4), minor bleeding (n = 2), nausea sensation (n = 2)
	TG: n = 59, 11 F, age 54y; CG: n = 59, 7 F, age 53y;				Daily cigarette consumption: Significant within-group effect—TG & CG (after treatment)	
	*Inclusion*: smoking>1 year and ≥10 cigarettes/day; ≥18 years; no addiction to other drugs					
Yeh et al. 2009 Taiwan[34]	79 randomized and 59 completed.	*Auricular electroacupuncture plus acupressure group* Acupoints: Endocrine, Lung, Mouth, Shenmen, Stomach, Tim mee; Manipulation: <60Hz frequency, 20 mins/session, Weekly; Vaccariae seeds acupressure 1 min/time, 3–5 times/day; 6 Wks	*Sham group* 5mm from the real acupoints; Same manipulation	N/A	Daily cigarette consumption: Significant within-group effect—TG & CG	N/A
	TG: n = 30, age 28y; CG: n = 29, age 27y;					
	*Inclusion*: smoking>1y and >1 cigarette/day; serum cotinine concentration >100ng/ml; no co-intervention for smoking					
Chae et al. 2010 Korea[35]	29 completed.	*Body acupuncture group* Acupoint: HT7; Manipulation: needle stimulation 30 seconds and withdrawn 20 minutes, 2 days	*Non-specific acupuncture group* Acupoint: LI10; Manipulation: blunted needle via a device, 2 days	N/A	Withdrawal symptoms: Significant between-group effect—TG>CG	None
	TG: n = 15; CG: n = 14;					
	*Inclusion*: >10 cigarettes/day; >18 years; male; no co-intervention for smoking; no addiction to other drugs					
Wing et al. 2010 Hong Kong[36]	70 randomized and 51 completed at Wk 3, Mo 3.	*Auricular acupressure group* Auricular acupoints: Brain, Lung, Mouth, Shenmen; Manipulation: beads pressed when feeling craving, ≥3 times/day, 3 Wks	*Non-specific acupuncture group* Acupoints: non-specific non-meridian points; Same manipulation	N/A	Daily cigarette consumption: Significant within-group effect—TG (after treatment, 3-Mo F/U), CG (after treatment)	Skin allergy (n = 3)
	TG: n = 38, 12 F, age 47y; CG: n = 32, 9 F, age 46y;					
	*Inclusion*: daily cigarette smoking; ≥18 years; no co-intervention for smoking					
Lambert et al. 2011 Singapore[30]	58 randomized and 55 completed.	*10mA body TEAS group* Acupoints: LI4, PC6, PC8, TE5; Manipulation: 1 sessions on Day 1, 3 sessions on Day 2 while abstaining from smoking, 26 hours	*1*. *i5mA TEAS group* (intermittent: 3min on and 7min off) *2*. *Sham TEAS group* (no electrical stimulation) Same acupoints/manipulation	N/A	Desire to smoke: Significant between-group effect—TG>both CGs if FTND≥5	Coughing, giddiness, finger/hand numbness (TG: 9; CG2: 3); runny nose (CG1: 3)
	TG: n = 21, 6 female, age 25y; CG1: n = 20, 7 female, age 24y; CG2: n = 17, 2 female, age 26y;					
	*Inclusion*: smoking>1-year and ≥15 cigarettes/day; FTND score≥4; no NRT within 3-Mo					
Fritz et al. 2013 USA[32]	125 randomized and 105 completed.	*Auricular TEAS group* Acupoints: Lung, Nicotine, Palate, Shenmen, Zero; Manipulation: acupuncturists administered. 80Hz frequency, 20 mins/session, Weekly, 5 Wks	*Sham group* Same acupoints/manipulation; No electrical stimulation	1-hour ‘stop-smoking’ class	Daily cigarette consumption, withdrawal symptoms: NS	15 reported (no detail)
	TG: n = 64, 16 F, age 56y; CG: n = 61, 14 F, age 55y;					
	*Inclusion*: ≥10 cigarettes/day; PHQ-9<20; urine cotinine≥200mg/ml; >19 years; no co-intervention for smoking					
Zhang et al. 2013 Australia[37]	43 randomized and 19, 12 completed at Wk 8, Mo 3.	*Specific auricular acupressure group* Acupoints: Hunger, Liver, Lung, Mouth, Shenmen; Manipulation: acupuncturists administered. Beads pressed ≥3 times/day when feeling craving, 8 Wks	*Nonspecific acupressure group* Acupoints: Clavicle, Helix 2, Occiput, Shoulder, Tooth; Same manipulation	N/A	Daily cigarette consumption, withdrawal symptoms, Smoking cessation rate: NS	Discomfort on ears (TG: 1; CG: 4); Headache, dizziness (CG: 1)
	TG: n = 20; 12 F, age 50y; CG: n = 23; 13 F, age 50y;					
	*Inclusion*: smoking>1-year and >10 cigarettes/day; >18 years; no co-intervention for smoking; no auricular acupuncture last year					
Baccetti et al. 2015 Italy[38]	477 randomized and 472, 447, 445 at Wk 5, Mo 6, Year 1.	*1*. *Body acupuncture*, *auricular acupressure plus psychological support group 2*. *Body acupuncture*, *auricular acupressure group* Acupoints: Pharmacopuncture: 1% lidocaine solution injected into LI20, auricular Zero; Plum-blossom needle: C7 to T5, 0.5, 1.5 and 3Cun from the vertebral spinous processes; Auricular acupressure: Shenmen; Manipulation: medical doctors administered, 5 Wks. Plum-blossom needle 3 times/session. Vaccaria seeds acupressure≥8 times/day when feeling craving, 30 min/session. Group 1 started after the 3rd psychological meeting, group 2 started immediately; *Psychological support group*: 1.5 hours/time, 9 times/5 Wks	*Sham body acupuncture*, *auricular acupressure plus psychological support group* Acupoints: Pharmacopuncture: 0.2cc lidocaine solution pricked below LI20 and Zero; Plum-blossom needle: C7 to T5, 2, 4 and 6 Cun from the vertebral spinous processes; Auricular acupressure: 0.5 cm from Shenmen; Manipulation: needles without tips for plum-blossom treatment	N/A	Smoking cessation rate: NS	Minor bleeding
	TG1: n = 159, 102 F, age 51y; TG2: n = 162, 103 F, age 50y; CG: n = 156, 100 F, age 48y;					
	*Inclusion*: smoking ≥1-year and ≥10 cigarettes/day; >18 years; Italian speaker; no co-intervention for smoking					
**Hypertension**						
Macklin et al. 2006 USA[39]	192 randomized and 188 completed at Wk 10, Mo 12.	*1*. *Individualized auricular-body acupuncture group* Acupoints: prescribed individually; *2*. *Standardized auricular-body acupuncture at preselected points group* Body acupoints: GB20, LI11, LR3, SP6, ST36; Auricular acupoints: Heart, Jiangyagou; Manipulation: acupuncturists administered. Neutral needle stimulation, 30 mins/session, Twice/Wk, 10 Wks	*Sham group* Body acupoints: 5 which are not acupoints; Auricular acupoints: Darwin’s tubercle, Ear lobe; Manipulation: no manipulation, 10 Wks	No antihypertensive medications	SBP, DBP: NS	Hypertensive urgencies (TG2: 2); congestive heart failure (CG: 1)
	TG1: n = 64, 30 F, age 57y; TG2: n = 64, 35 F, age 56y; CG: n = 64, 35 F, age 53y;					
	*Inclusion*: stable BP: 140/90-179/109mmHg after 8–31 days suspension of antihypertensive medications; ≥18 years; no acupuncture within 6-Mo					
Flachskampf et al. 2007 Germany[48]	160 randomized and 140, 140, 135, 133 completed at Day 3, Wk 6, Mos 3, 6.	*Body acupuncture group* Acupoints: prescribed individually; Manipulation: acupuncturists administered. 20 mins/session, 22 sessions/6 Wks	*Non-specific acupuncture group* Acupoints: GB31, GB32, GB34, SI7, etc.; Same manipulation	Maintain antihypertensive medication before study	SBP, DBP: Significant within-group effect—TG (after treatment); Significant between-group effect—TG>CG (after treatment, 3-day F/U)	Pain, atrial fibrillation (TG); pain (CG)
	TG: n = 72, 33 F, age 59y; CG: n = 68, 40 F, age 58y;					
	*Inclusion*: stable BP: 140/90-220/115mmHg; 45–75 years					
Yin et al. 2007 Korea[44]	41 randomized and 30 completed.	*Body acupuncture plus exercise group* Acupoints: (1) BL25, LI11, ST36 for tonification of the large intestine meridian; (2) BL13, LU9, SP3 for the lung meridian; (3) KI2, KI7, RN4 for the kidney meridian; (4) DU14, GB20, LI1 for the bladder meridian; Manipulation: physicians administered. Needle stimulation until Deqi. 17 sessions, 8 Wks; *Exercise*: breathing exercise 10 mins/day, walking 30 mins/day, 8 Wks	*Sham acupuncture plus exercise group* Same acupoints; Manipulation: until Deqi, withdrawn immediately	Maintain antihypertensive medication before study	SBP, DBP: Significant within-group effect—TG; Significant between-group effect—TG>CG (4^th^-8^th^ Wk)	Bleeding on needle removal (TG: 8)
	TG: n = 15, 11 F; CG: n = 15, 10 F;					
	*Inclusion*: SBP: 120-179mmHg or DBP: 80-99mmHg; SBP: 140-179mmHg or DBP: 90-99mmHg with antihypertensive medications					
Zhang et al. 2008 USA[41]	47 randomized and 45 completed (14 F, age 25y).	*Laser body acupuncture group* Acupoints: LI4, LI11; Manipulation: 40 infrared laser, 10kHz frequency, 8 mins/session, twice/Wk, 12 sessions	*Sham group* Same acupoints/manipulation; No activated laser beam	N/A	SBP, DBP: Significant within-group effect—TG	N/A
	TG: n = 23; CG: n = 22;					
	*Inclusion*: SBP: 125-160mmHg and/or DBP: 81-110mmHg; no previous laser therapy use					
Zhang et al. 2009 USA[42]	27 randomized and completed (age 25y).	*Body electroacupuncture group* Acupoints: LI4, LI11; Manipulation: 100Hz frequency, 15 mins/acupoint/session, Twice/Wk, 5 Wks	*Sham group* Same acupoints/manipulation; No electric stimulation	Maintain diet, exercise, antihypertensive medications	SBP: Significant within-group effect—TG	N/A
	TG: n = 13; 5 F; CG: n = 14; 5 F;					
	*Inclusion*: SBP: 120-165mmHg; DBP: 80-110mmHg; no co-intervention for hypertension					
Kim et al. 2012 Korea[40]	33 randomized and 28 completed.	*Body acupuncture group* Acupoints: PC6, ST36; Manipulation: Korean medicine practitioners administered. Needle stimulation until Deqi. 20 mins/session, twice/Wk, 8 Wks	*Sham group* Acupoints: 1cm from PC6, ST36; Manipulation: no manipulation	No antihypertensive medications during the treatment	Nighttime DBP: Significant within-group effect—TG; Significant between-group effect—TG>CG	Slight injection-site pain, small bleeding (TG)
	TG: n = 12; CG: n = 16;					
	*Inclusion*: SBP: 140-159mmHg or DBP: 90-99mmHg; 18–70 years; no previous antihypertensive medication use					
Chen et al. 2013 China[46]	32 randomized and 30 completed (16 F, age 57y).	*Body acupuncture group* Acupoints: DU20, DU23, EX-HN1, LI4, LI11, LR3, PC6, SP6, ST9, ST36; Manipulation: acupuncturists administered. Needle stimulation until Deqi. 30 mins/session, 5 days	*No intervention group*	Maintain antihypertensive medications before study	SBP, DBP: NS	N/A
	TG: n = 15; CG: n = 15;					
	*Inclusion*: SBP≥140mmHg and DBP≥90mmHg without medication; 30–75 years; no acupuncture last year					
Sriloy et al. 2015 India[49]	46 randomized and 38 completed.	*Auricular-body acupuncture group* Body acupoints: DU20, HT7, LR3, ST36; Auricular acupoint: Shenmen; Manipulation: naturopaths administered. Needle stimulation until Deqi. 20 mins/session	*Slow breathing group*	N/A	DBP: Significant within-group effect—TG;	N/A
	TG: n = 19, 4 F, age 48y; CG: n = 19, 6 F, age 50y;				SBP: Significant within-group effect—CG	
	*Inclusion*: diagnosed hypertension≥3 years; 35–60 years; no previous acupuncture use					
Li et al. 2015 USA[43]	65 randomized and 64, 20 completed at Wk 8, Mo 3.	*Body electroacupuncture group* Acupoints: PC5, PC6, ST36, ST37; Manipulation: 2-5Hz frequency, 30 mins/session, Weekly, 8 Wks	*Non-specific acupuncture group* Acupoints: LI6, LI7, GB37, GB39; Same manipulation	N/A	SBP: Significant between-group effect—TG>CG (after treatment, 1-Mo F/U);	None
	TG: n = 33, 17 F, age 58y; CG: n = 32, 18 F, age 54y;				DBP: NS	
	*Inclusion*: SBP/DBP≥140-180/90–99mmHg; no antihypertensive medications within 3-day before enrolment					
Liu et al. 2015 Korea[45]	30 randomized and 26 completed at Wks 8, 12.	*Body acupuncture group* Acupoints: LI11, LR3, PC6, SP4, ST36; Manipulation: needle stimulation until Deqi. 20 mins/session, twice/Wk, 8 Wks	*No intervention group*	N/A	SBP: Significant within-group effect—TG (after treatment, 4Wk F/U);	None
	TG: n = 15, 12 F, age 49y; CG: n = 15, 11 F, age 53y;				DBP: Significant within-group effect—TG (after treatment);	
	*Inclusion*: SBP: 120–159 mmHg or DBP: 80–99 mmHg; 20–65 years; no co-intervention for hypertension; no acupuncture within 6-Mo				SBP, DBP: Significant between-group effect—TG> CG (after treatment, 4Wk F/U)	
Lin et al. 2016 Taiwan[50]	80 randomized and completed.	*Body acupressure group* Acupoints: LR3; Manipulation: press 5 seconds and release 1 second, 30 times	*Sham group* Acupoints: 1inch from LR3; Same manipulation	N/A	SBP, DBP: Significant within-group effect—TG; Significant between-group effect—TG>CG (immediately, 15-/30-min after acupressure)	N/A
	TG: n = 40, 20 F, age 59y; CG: n = 40, 20 F, age 63y;					
	*Inclusion*: SBP: 150-180mmHg; 40–75 years; no antihypertensive medication within 2h before enrolment					
Zhan et al. 2016 China[47]	174 patients randomized and completed.	*1*. *Laser body acupuncture plus musical group 2*. *Laser body acupuncture group* Acupoints: LI11, LR3 for liver fire hyperactivity syndrome; KI3, SP6 for yin-deficiency and yang-hyperactivity syndrome; ST36, ST40 for excessive phlegm-dampness syndrome; KI3, RN4 for yin-yang deficiency syndrome; Manipulation: 30 mins/session, daily; 30 days. Group 1: listening to music while laser stimulation (650nm wavelength, 0.5cm diameter). Group 2: needle stimulation until Deqi	*Starch tablets* 25 mg/tablet, 1 tablet/time, 3 times/day before meal, 30 days	N/A	SBP, DBP: Significant within-group effect—TG1 & TG2; Significant between-group effect—TG1>CG; TG2>CG	N/A
	TG1: n = 58, 33 F, age 50y; TG2: n = 58, 31 F, age 50y; CG: n = 58, 34 F, age 50y;					
	*Inclusion*: SBP: 140-159mmHg and/or DBP: 90-99mmHg; 25–69 years; BMI: 18–30 kg/m^2^; no previous antihypertensive drugs (or stopped ≥2 Wks)					
**Obesity**						
Richards & Marley 1998 Australia[51]	60 randomized and 50 completed.	*Auricular acupuncture group* Acupoints: Shenmen, Stomach; Manipulation: acuSlim device. 15–20 mins/session, twice/daily, 4 Wks	*Sham group* Acupoints: thumb (no acupoints); Same manipulation	Maintain diet	Weight loss ≥2kg, suppression of appetite: Significant between-group effect—TG>CG	N/A
	TG: n = 28, age 44y; CG: n = 32, age 43y;					
	*Inclusion*: BW<120kg; >18 years; stable BW≥3 Mos; no co-intervention for obesity					
Mazzoni et al. 1999 Italy[52]	40 randomized and 22 completed.	*Auricular-body acupuncture moxibustion group* Acupoints: Sessions 1–3, Moxibustion: BL14, BL15, BL20; body acupoints: DU20, HT9, RN14, RN17, SP1; auricular acupoints: Hunger, Stomach; Sessions 4–8, body acupoints: BL10, BL60, LR13, RN12, RN15, SP7, ST36; auricular acupoints: Hunger, Stomach; Sessions 9–12, body acupoints: DU20, RN14, SJ10, ST40, ST44; auricular acupoints: Shenmen; Manipulation: acupuncturist administered. Weekly, 12 Wks	*Sham group* Body acupoints: 3mm from the real acupoints; Manipulation: superficial insertion (3-5mm), 12 Wks	No medications for obesity; restricted saturated fats and snacks; daily abdominal self-massage 30–45 minutes	BMI, suppression of appetite: NS	N/A
	TG: n = 20, 16 F, age 37y; CG: n = 20, 17 F, age 40y;					
	*Inclusion*: BMI>30kg/m^2^; 18–60 years; no other disorders or treatments determining weigh gain					
Wei & Liu 2004 China[53]	195 randomized and completed (187 F, age 36y).	*Auricular-body acupuncture group* Body acupuncture: Acupoints: LI4, LI11, ST36, ST37, ST44 for excess-heat in stomach and intestines syndrome (syndrome 1); RN6, RN12, SP6, SP9, ST36, ST40 for damp retention due to spleen deficiency syndrome (syndrome 2); BL23, KI6, RN4, SJ6 for kidney qi insufficiency syndrome (syndrome 3); BL18, GB43, LR3, LR8 for liver qi stagnation syndrome (syndrome 4); Auricular acupuncture: Acupoints: Endocrine, Hunger, Lung, Shenmen (syndrome 1); Endocrine, Lung, Spleen, Stomach (syndrome 2); Endocrine, Kidney, Lung, Triple energizers (syndrome 3); Endocrine, Liver, Shenmen (syndrome 4); Manipulation: 30 mins/session, every two-day, 12 sessions	*1*. *Body acupuncture group* Same with the body acupuncture treatment; *2*. *Auricular acupuncture group* Same with the auricular acupuncture treatment; Same manipulation	N/A	Weight loss≥3kg: Significant between-group effect—TG>CG1; TG>CG2	N/A
	TG: n = 76; CG1: n = 64; CG2: n = 55;					
	*Inclusion*: BMI>25kg/m^2^ (F)/26kg/m^2^ (M); no co-intervention for obesity					
Hsu et al. 2005 Taiwan[54]	72 randomized and 63 completed.	*Body electroacupuncture group* Acupoints: KI14, RN6, RN9, SP6, ST26, ST28, ST40; Manipulation: 42Hz frequency to maximal tolerable intensity. Needle stimulation until Deqi. 40 mins/session, twice/Wk, 6 Wks	*1*. *Sit-up exercises group* 10 times/day, 6 Wks *2*. *No intervention group*	Maintain diet	BW, BMI, WC: Significant between-group effect—TG>CG1; TG>CG2	Mild ecchymosis (n = 3); abdominal discomfort (n = 1)
	TG: n = 22, age 40y; CG1: n = 20, age 41y; CG2: n = 21, age 41y;					
	*Inclusion*: WC>90cm; female; BMI>30kg/m^2^; 16–65 years; no co-intervention for obesity within 3-Mo and the study					
Elder et al. 2007 USA[55]	92 randomized and 73 completed.	*1*. *Qigong group* Shaking (5 minutes), Movements (18 minutes), harvest the energy method (5 minutes), 24 Wks; *2*. *Body Tapas acupressure technique group* Acupoints: BL1, EX-HN3, GB21; Manipulation: acupuncturists administered. 1 min/session, daily, 24 Wks	*Self-directed support* Written materials and maintenance support groups, 24 Wks	N/A	Weight loss of 2.8kg: Significant between-group effect—TG2>TG1	None
	TG1: n = 31, 26 F, age 48y; TG2: n = 30, 27 F, age 48y; CG: n = 31, 26 F, age 46y;					
	*Inclusion*: BMI: 25–35 kg/m^2^ (F)/25–40 kg/m^2^ (M); weight change<10 pounds within 6-Mo; 18–80 years; no co-intervention for obesity within 6-Mo; no previous complementary medicine use; no other disorders determining weigh gain; alcohol<21 drinks/Wk					
Hsieh 2007 Taiwan[56]	70 randomized and 55 completed.	*Auricular acupressure group* Acupoints: Endocrine, Mouth, Shenmen, Small intestine, Stomach; Manipulation: Japanese magnetic pearl acupressure. 10 mins/session, Weekly, 8 Wks	*Sham group* Same acupoints; Acupressure tape only	Education on low-calorie diet; maintain physical activity	BMI: Significant within-group effect—TG	N/A
	TG: n = 27, 24 F; CG: n = 28, 26 F;					
	*Inclusion*: BMI≥23kg/m^2^; 18–20 years; Asian ethics					
Yeh & Yeh 2008 Taiwan[57]	38 randomized and completed.	*Auricular acupressure group* Acupoints: Endocrine, Mouth, Shenmen, Small intestine, Stomach; Manipulation: beads acupressure before meals, 15 mins/session, Weekly, 9 Wks	*No intervention group*	Maintain diet, physical activity	WC, HC: Normal weigh participants Significant within-group effect—TG & CG; Obese participants: NS	None
	TG: n = 19, 16 F, age 33y; CG: n = 19, 16 F, age 33y;					
	*Inclusion*: BMI≥27 kg/m^2^ (obese)/<27 kg/m^2^ (normal weight); 22–50 years; no acupuncture for obesity within 1-Mo					
Nourshahi et al. 2009 Iran[58]	27 randomized and completed.	*1*. *Exercise plus low-calorie diet group* 3 sessions/Wk, 8 Wks; *2*. *Auricular-body acupuncture*, *exercise plus low-calorie diet group* Auricular acupoints: Hunger, Shenmen; Body acupoint: ST40; Manipulation: acupuncturists administered. 8 Wks. Body acupuncture 20 mins/session; Lentil seeds auricular acupressure 10 times/30 mins before meals and whenever feeling hungry	*No intervention group*	N/A	BMI, fat mass: Significant between-group effect—TG1>CG; TG2>CG	N/A
	TG1: age 42y; TG2: age 40y; CG: age 37y;					
	*Inclusion*: fat mass>30%; female					
Hsu et al. 2009 Taiwan[59]	60 randomized and 45 completed.	*Auricular acupuncture group* Acupoints: Endocrine, Hunger, Shenmen, Stomach; Manipulation: acupuncturists administered. Twice/Wks, 6 Wks	*Sham group* Same acupoints/manipulation; Needles without tips	Maintain diet	BW, BMI, WC: NS	Minor inflammation (TG: 1); tenderness (TG: 7; CG: 2)
	TG: n = 23, age 40y; CG: n = 22, age 39y;					
	*Inclusion*: BMI >27kg/m^2^; female; 16–65 years; no co-intervention for obesity within 3-Mo and the study					
Hsieh et al. 2010, 2011, 2012 Taiwan[60,61,65]	84 randomized and 68 completed.	*1*. *Auricular acupressure (Japanese magnetic pearl) group 2*. *Auricular acupressure (Vaccaria seeds) group* Auricular acupoints: Endocrine, Mouth, Shenmen, Small intestine, Stomach; Manipulation: 10 mins/session/Wk, 8 Wks	*Sham group* Same acupoints/manipulation; Acupressure tape only	Education on low-calorie diet; maintain physical activity	BMI: Significant within-group effect—TG1 & TG2;	N/A
	TG1: n = 27, 24 F; TG2: n = 29, 26 F; CG: n = 28, 26 F;				BW, WC: Significant within-group effect—TG1, TG2, & CG; Significant between-group effect—TG2>TG1;	
	*Inclusion*: WC≥80cm (F)/90cm (M); BMI>23kg/m^2^; 18–20 years				Waist-to-hip ratio: Significant within-group effect—TG1 & TG2	
Rerksuppaphol & Rerksuppaphol 2011 Thailand[62]	45 randomized and completed.	*Body TEAS group* Acupoints: RN4, RN6, RN10, RN12, SP15, ST25, ST28; Manipulation: acupuncturists administered. Electrodes with 40Hz frequency, 30 mins/session, twice/Wk, 8 Wks	*Body electroacupuncture group* Same acupoints; Manipulation: disposable needles with 40Hz frequency, 30 mins/session, twice/Wk, 8 Wks	Maintain diet, exercise, medication for obesity	Weight loss, BMI: Significant within-group effect—TG	None
	TG: n = 23, age 34y; CG: n = 22, age 33y;					
	*Inclusion*: BMI>23kg/m^2^; WC>80cm; female; >15 years; no co-intervention for obesity					
Rerksuppaphol 2012 Thailand[63]	40 randomized and 29 completed.	*Body TEAS plus auricular acupressure group* Body acupoints: RN4, RN6, RN10, RN12, SP15, ST25, ST28; Auricular acupoints: Shenmen, Hungry, Stomach; Manipulation: acupuncturists administered. Electrodes with 40Hz frequency, 30 mins/session, twice/Wk, 8 Wks	*Auricular acupressure group* Same acupoints; Manipulation: magnetic pellets acupressure. Self-stimulation, 10 times/session, 3 sessions/day before meals, 8 Wks	Maintain diet, exercise	BW, BMI, WC, waist-to-hip ratio: Significant within-group effect—TG;	N/A
	TG: n = 20, age 41y; CG: n = 20, age 32y;				BW, BMI: Significant between-group effect—TG>CG	
	*Inclusion*: BMI>23kg/m^2^; female; >15 years; no other medications for obesity					
Lien et al. 2012 Taiwan[64]	90 randomized and 71 completed.	*1*. *Auricular acupuncture group 2*. *Auricular acupressure group* Acupoints: Endocrine, Hunger, Shenmen, Stomach; Manipulation: acupuncturists administered. Beads acupressure 3 sessions/Wk, 4 Wks	*Sham acupuncture group* Same acupoints/manipulation; Needles without tips	Maintain diet, lifestyle	BW, BMI, WC: Significant within-group effect—TG1 & TG2	Dizziness (TG1: 1).
	TG1: n = 24, age 39y; TG2: n = 24, age 42y; CG: n = 23, age 41y;					
	*Inclusion*: BMI≥27kg/m^2^; female; 16–60 years; no co-intervention for obesity within 2-Mo; no previous auricular acupuncture					
Darbandi et al. 2012 Iran[66]	90 randomized and 86, 84 completed at Wk 6, Mo 2.	*Auricular acupressure group* Acupoints: Centre of ear, Hunger, Mouth, Sanjiao, Shenmen, Stomach; Manipulation: acupuncturists administered. Vaccaria seed acupressure before meals, 6 Wks	*Non-specific acupressure group* Acupoints: Hip, Nose, Oesophagus, Spleen; Manipulation: plasters without seeds, 6 Wks	Low-calorie diet	BW, BMI: Significant within-group effect—TG & CG (after treatment)	None
	TG: n = 43, 37 F, age 38y; CG: n = 43, 37 F, age 38y;					
	*Inclusion*: BMI: 25-45kg/m^2^; 18–55 years; no co-intervention for obesity; no medications for obesity within 3-Mo					
Abdi et al. 2012 Iran[67]	196 randomized and 161 completed at Wks 6, 12.	*Body acupuncture group* Acupoints: GB28, RN4, RN9, RN12, SP6, ST25. For excess syndromes, LI11, ST40 added; For deficiency syndromes, RN6, SP9 added; Manipulation: acupuncturists administered. Needle stimulation until Deqi. GB28, ST25 applied with electricity at 30-40Hz frequency. 20 mins/session, twice/Wk, 6 Wks	*Sham group* Acupoints: on the RN meridian, 0.3cm from the real acupoints; Manipulation: superficial needling. Disconnected electric lines	Low-calorie diet	BW, BMI, HC: Significant within-group effect—TG & CG (after treatment, 6Wk F/U);	None
	TG: n = 79, age 37y; CG: n = 82, age 37y;				WC: Significant within-group effect—TG (after treatment, 6Wk F/U), CG (after treatment)	
	*Inclusion*: same with Darbandi et al. 2012					
He et al. 2012 China[68]	60 randomized and completed (age 34y).	*Auricular acupressure plus exercise group* Acupoints: Endocrine, Hunger, Large intestine, Shenmen, Spleen, Stomach; Manipulation: vaccariae seed acupressure, 10 seconds/time, 3 times/day, 4 Wks	*Exercise group* Heart rates at 120–150 beats/min, 1 hour/day, 4 Wks	Low-calorie diet; no food after 8PM	BW, BMI, WC: Significant within-group effect—TG & CG;	N/A
	TG: n = 30; CG: n = 30;				BW: Significant between-group effect—TG>CG	
	*Inclusion*: BMI≥25kg/m^2^; WC≥80cm; female; 18–60 years; no medical/drug use for obesity within 2-Mo					
Guo et al. 2014 China[69]	64 randomized and 61 completed (32 F, age 37y).	*Body electroacupuncture plus diet group* Acupoints: RN4, RN12, SP6, SP9, ST25, ST36, ST40; Manipulation: 1400kcal/diet. 2Hz frequency stimulation, 30 mins/session, daily, 45 days	*Diet group* 1400kcal/diet. 45 days	N/A	BW: Significant within-group effect—TG & CG	N/A
	TG: n = 32; CG: n = 32;					
	*Inclusion*: BMI ≥28kg/m^2^ (obese); >18 years					
Wu et al. 2014 China[70]	72 randomized and 65 completed.	*Body acupuncture group* Acupoints: SP4, SP6, SP8, SP9, SP10, SP14, SP15; Manipulation: acupuncture at 9-11AM. Needle stimulation until Deqi. 30 mins/session, daily, 30 days	*Sham group* Same acupoints; Manipulation: acupuncture at any time beyond 9-11AM	N/A	BW, BMI, WC, HC: Significant within-group effect—TG & CG	N/A
	TG: n = 36, age 28y; CG: n = 36, age 28y;					
	*Inclusion*: BMI≥25kg/m^2^; 18–65 years; WC≥80cm (F)/90cm (M); TCM syndrome of spleen deficiency and exuberant dampness; no drug within 6-Mo; no co-intervention for obesity					
Kim et al. 2014 Korea[71]	58 randomized and 49 completed.	*Auricular acupressure group* Acupoints: Endocrine, Mouth, Shenmen, Small intestine, Stomach; Manipulation: salba seeds acupressure 30 minutes before meals, 5 sec/point, 10 times/point, 3 times/day, 1 Mo	No information	N/A	BW, BMI: Significant within-group effect—TG & CG; Significant between-group effect—TG>CG	N/A
	TG: n = 25, age 21y; CG: n = 24, age 21y;					
	*Inclusion*: BMI≥25kg/m^2^; female; no previous medication; no co-intervention for obesity					
Yeo et al. 2014 Korea[72]	91 randomized and 58 completed.	*Auricular acupuncture group 1* Acupoints: Endocrine, Hunger, Shenmen, Spleen, Stomach; *Auricular acupuncture group 2* Acupoint: Hunger; Manipulation: Korean medicine practitioners administered. 8 Wks	*Sham group* Acupoints: same with TG1; Manipulation: needles removed immediately after insertion, 8 Wks	Low-calorie diet; no extra exercise	BW, BMI: Significant between-group effect—TG1>CG; TG2>CG;	N/A
	TG1: n = 31, 25 F, age 35y; TG2: n = 30, 25 F, age 39y; CG: n = 30, 25 F, age 43y;				WC: Significant between-group effect—TG1>CG	
	*Inclusion*: BMI≥23kg/m^2^; >19 years; daily ambulatory time<2h; stable weight; no co-intervention for obesity within 6-Mo; no addiction to alcohol					
Schukro et al. 2014 Austria[73]	56 randomized and 45, 42 completed at Wks 6, 10.	*Auricular electroacupuncture group* Acupoints: Colon, Hunger, Stomach; Manipulation: 1Hz frequency. 7AM-11AM, 4 days/Wk, 6 Wks	*Sham group* Same acupoints/manipulation; No electric stimulation	N/A	BW, BMI: Significant between-group effect—TG>CG (after treatment, 4Wk F/U)	Skin irritations (n = 8)
	TG: n = 28, age 54y; CG: n = 28, age 50y;					
	*Inclusion*: BMI>25kg/m^2^; female; >18 years; no previous acupuncture use					
Darbandi et al. 2014 Iran[74]	80 randomized and completed.	*1*. *Body electroacupuncture group* Acupoints: same with Abdi et al 2012; Manipulation: acupuncturists administered. 30–40Hz frequency at a maximal tolerable intensity, 20 mins/session, twice/Wk, 6 Wks; *2*. *Auricular electroacupuncture group* Acupoints: Center of ear, Hungry, Mouth, Sanjiao, Shenmen, Stomach; Manipulation: vaccaria seeds acupressure before meals, twice/Wk, 6 Wks	*1*. *Sham body electroacupuncture group* Acupoints: 0.5cun from the real acupoints; Manipulation: superficial insertion. No electric stimulation; *2*. *Sham auricular electroacupuncture group* Acupoints: Esophagus, Hip, Nose, Spleen; Manipulation: plasters without seeds	Low-calorie diet	BMI: Significant between-group effect—TG1>CGs1, 2; TG2>CGs1, 2;	None
	TG1: n = 20, age 38y; TG2: n = 20, age 39y; CG1: n = 20, age 38y; CG2: n = 20, age 38y;				HC: Significant between-group effect—TG1>CGs1, 2; TG2>CGs1, 2; TG2>TG1;	
	*Inclusion*: BMI: 30–40kg/m^2^; male; 18–50 years; no medications for obesity within 3-Mo				WC: Significant between-group effect—TG1>CGs1, 2; TG2>CG2; TG1>TG2	
Yeh et al. 2015 Taiwan[75]	134 randomized and 70 completed (35 F).	*Auricular electroacupuncture & acupressure group* Acupoints: Endocrine, Hunger, Shenmen, Stomach; Manipulation: researchers administered. 10 Wks. Needle stimulation until Deqi. 2-100Hz frequency, 20 mins/session, Weekly; Vaccariae seeds acupressure 1 min/point, 4 times/day	*Non-specific acupuncture group* Acupoints: Ankle, Clavicle, Elbow, Shoulder; Same manipulation	Nutrition counselling	BMI: Significant within-group effect—TG & CG	N/A
	TG: n = 36, age 30y; CG: n = 34, age 33y;					
	*Inclusion*: BMI≥27kg/m^2^; WC≥80cm (F)/90cm (M); 18–50 years; no medication/surgery use for obesity within 3-Mo					
He et al. 2015 China[76]	56 randomized and completed.	*Body acupuncture plus massage group* Acupoints: LI11, RN6, RN12, SJ6, SP6, SP15, ST21, ST25, ST36; Manipulation: acupuncturists administered. Needle stimulation until Deqi. 30 mins/session, daily, 21 days; *Massage*: stomach Meridian, Ren Meridian, Dai Meridian (abdomen), 25 mins/session, daily, 21 days	*Body acupuncture group* Same acupoints/manipulation	Maintain diet; no physical training or exercise	BW, BMI: Significant within-group effect—TG & CG	N/A
	*Inclusion*: BMI≥25kg/m^2^; female; no previous drug use					
Jiao et al. 2015 China[77]	48 randomized and completed.	*Body electroacupuncture plus running group* Acupoints: SP6, ST36; Manipulation: 50Hz frequency, 45 mins/session, daily, 6 Wks; *Running*: 45 mins/day, 6 Wks	*Running group* 45 mins/day, 6 Wks	No overeating	Fat mass: NS	N/A
	TG: n = 24, age 35y; CG: n = 24, age 36y;					
	*Inclusion*: BMI>28kg/m^2^; male					

^a^Add-on strategy of all the intervention groups.

Wk: Week; TG: treatment group; CG: Control group; F: Female; Age, mean age; Mo: Month; DSM-III-R, Diagnostic and Statistical Manual of Mental Disorders IIIR; NS, not statistically significant; N/A, not available; F/U: follow-up; ICD-10, International Statistical Classification of Diseases and Related Health Problems 10th Revision; DSM-IV, Diagnostic and Statistical Manual of Mental Disorders 4^th^ed; NRT, nicotine replacement therapy; FTND, Fagerstrom Test for Nicotine Dependence; TEAS, transcutaneous electric acupoint stimulation; PHQ-9, 9-item Patient Health Questionnaire; BP, blood pressure; SBP, systolic blood pressure; DBP, diastolic blood pressure; BW, body weight; BMI, body mass index; WC, waist circumference; HC, hip circumference; M: male.

### Data syntheses

Cochrane RevMan version 5.3 software was employed to conduct meta-analysis of the outcome measures and heterogeneity was determined using I^2^ statistic [15]. The meta-analysis included all studies where acupuncture was employed with or without co-interventions, provided that such intervention was given to all groups. However, meta-analyses were conducted only if at least two RCTs were available exploring a specific outcome of a risk factor. Acupuncture approaches shown in the meta-analysis include needle acupuncture (body, aural region, electroacupuncture), laser acupuncture, and acupressure. Analyses were performed separately for type of experimental interventions (acupuncture, acupressure, laser acupuncture, or the combination of acupuncture and acupressure) according to the RCT design. Random effects model (Mantel-Haenszel for dichotomous/categorical variables and inverse variance for continuous variables) was used to calculate mean differences (MD), standardized mean differences (SMD), or risk ratios (RR), and 95% confidence intervals (CI) were reported. Sensitivity analyses were used to test the robustness of statistically significant results for RCTs with low risk versus high risk of bias for the domains selection bias and performance/detection bias. Effects sizes of acupuncture compared to other interventions were shown in Table 3.

**Table 3:** Effect sizes of acupuncture in comparison to sham acupuncture or no treatment.

Outcome	RCT number	Participant number		Heterogeneity (I^2^;Chi^2^;p)	Subgroup difference (95% confidence interval)	p (sub-group effect)
		Experimental group	Control group			
Smoking-dependence risk factor—Daily cigarette consumption						
Acupressure VS Sham intervention	2 [36,37]	58	55	0%;0.45;0.50	MD = -2.75 cigarette/day (-5.33, -0.17)	0.04
Smoking-dependence risk factor—Smoking withdrawal symptoms						
Acupuncture VS Sham intervention	3 [26,33,35]	89	89	90%;19.8;<0.001	SMD = -0.95 (-2.17,0.26)	0.12
Smoking-dependence risk factor—Smoking cessation rate (short-term)						
Acupuncture VS Sham intervention	3 [26,27,33]	205	205	0%;0.70;0.71	RR = 1.11 (0.85, 1.46)	0.44
Acupressure VS Sham intervention	2 [36,37]	58	55	0%;0.19;0.66	RR = 0.39 (0.08, 1.96)	0.26
Acupuncture plus acupressure VS Sham intervention	2 [23/24,38]	179	180	66%;2.96;0.09	RR = 2.51 (0.26, 24.24)	0.43
Smoking-dependence risk factor—Smoking cessation rate (long-term)						
Acupuncture VS Sham intervention	2 [26,33]	51	49	0%;0.52;0.47	RR = 1.13 (0.40, 3.21)	0.82
Acupressure VS Sham intervention	2 [36,37]	49	40	0%;0;0.95	RR = 2.43 (0.40, 14.66)	0.33
Acupuncture plus acupressure VS Sham intervention	2 [24,38]	164	170	22%;1.28;0.26	RR = 1.97 (0.67, 5.80)	0.22
Hypertension risk factor—Systolic blood pressure						
Acupuncture VS Sham intervention	2 [40,48]	84	84	78%;4.59;0.03	MD = -0.54 mmHg (-10.69, 9.60)	0.92
Hypertension risk factor—Diastolic blood pressure						
Acupuncture VS Sham intervention	2 [40,48]	84	84	0%;0.89;0.35	MD = -1.38 mmHg (-4.06, 1.31)	0.32
Obesity risk factor—Body weight						
Acupuncture VS No treatment	2 [54,69]	54	53	50%;1.99;0.16	MD = -1.12 kg (-5.51, 3.27)	0.62
Acupressure VS No treatment	2 [57,71]	44	43	32%;1.47;0.23	MD = -2.87 kg (-6.47, 0.74)	0.12
Acupuncture VS Sham intervention	4 [59,64,67,72]	157	157	0%;0.73;0.87	MD = -2.66 kg (-6.05, 0.72)	0.12
Acupressure VS Sham intervention	2 [64,66]	67	66	0%;0.41;0.52	MD = -1.01 kg (-4.55, 2.52)	0.57
Obesity risk factor—Body mass index						
Acupressure VS No treatment	2 [57,71]	44	43	49%;1.95;0.16	MD = -0.41 kg/m^2^ (-1.56, 0.73)	0.48
Acupuncture VS Sham intervention	5 [59,64,67,72,74]	177	177	18%;4.88;0.30	MD = 0.12 kg/m^2^ (-0.88, 1.13)	0.81
Acupressure VS Sham intervention	2 [64,66]	67	66	0%;0.26;0.61	MD = -0.44 kg/m^2^ (-1.65, 0.78)	0.48
Obesity risk factor—Waist circumference						
Acupuncture VS Sham intervention	5 [59,64,67,72,74]	177	177	0%;1.61;0.81	MD = -2.79 cm (-4.13, -1.46)	<0.001

^a^MD: Mean difference. SMD: standardized mean difference; RR: risk ratio; I^2^: the percentage of variation across studies that is due to heterogeneity; Chi^2^: chi-square test.

### Quality assessment

Two authors (DS and WP) independently assessed the risk of bias of all included studies using the Cochrane Risk of Bias Tool for selection bias (random sequence generation and allocation concealment), performance bias (blinding of participants and personnel), detection bias (blinding of outcome assessment), attrition bias (incomplete outcome data), reporting bias (selective outcome reporting), and other bias (Table 4). Disagreements were assessed by a third author. It is worth noting that, due to methodological reasons and the uniqueness of acupuncture treatments, it is not feasible to blind the acupuncturist in acupuncture RCTs. Therefore, we adopted the domain of performance bias and only focused on adequate participant blinding.

**Table 4:** Risk of bias of the included studies using the Cochrane Risk of Bias Tool.

Reference	Risk factor	Random sequence generation	Allocation concealment	Blinding of participants and personnel^a^	Blinding of outcome assessment	Incomplete outcome data	Selective reporting	Other bias
Rampes et al., 1997, UK [21]	Alcohol	Low risk	Low risk	High risk	Low risk	Low risk	Low risk	Low risk
Sapir-Weise et al., 1999, Sweden [20]	Alcohol	Unclear	Low risk	Low risk	Low risk	Low risk	Low risk	Unclear
Bullock et al., 2002, USA [22]	Alcohol	Low risk	High risk	High risk	Low risk	High risk	Low risk	Unclear
Karst et al., 2002, Germany [19]	Alcohol	Unclear	Unclear	Low risk	Low risk	Unclear	Low risk	Unclear
Trumpler et al., 2003, Switzerland [18]	Alcohol	Low risk	Low risk	High risk	Low risk	Low risk	Low risk	High risk
Kunz et al., 2007, Germany [17]	Alcohol	Unclear	Unclear	High risk	Unclear	High risk	Low risk	High risk
Lee et al., 2015, Korea [16]	Alcohol	Low risk	High risk	Low risk	Low risk	Low risk	Low risk	Unclear
He et al., 1997, Norway [23,24]	Smoking	Low risk	High risk	Low risk	Unclear	Low risk	Low risk	Low risk
Waite & Clough, 1998, UK [25]	Smoking	Unclear	Unclear	Low risk	Unclear	High risk	Low risk	Low risk
White et al., 1998, UK [26]	Smoking	Low risk	Low risk	Low risk	Low risk	High risk	Low risk	Low risk
Georgiou et al., 1998, UK [27]	Smoking	Low risk	High risk	High risk	Low risk	High risk	Low risk	Low risk
Cai et al., 2000, Singapore [29]	Smoking	Low risk	High risk	Low risk	Low risk	High risk	Low risk	Low risk
Bier et al., 2002, USA [31]	Smoking	Low risk	High risk	Low risk	Low risk	High risk	High risk	Low risk
White et al., 2007, UK [28]	Smoking	Low risk	Low risk	High risk	High risk	High risk	Low risk	Unclear
Wu et al., 2007, Taiwan [33]	Smoking	Low risk	High risk	Unclear	Unclear	Low risk	Low risk	High risk
Yeh et al., 2009, Taiwan [34]	Smoking	Unclear	Unclear	Unclear	Unclear	Unclear	High risk	Low risk
Chae et al., 2010, Korea [35]	Smoking	Low risk	High risk	Unclear	Unclear	Unclear	Low risk	Low risk
Wing et al., 2010, Hong Kong [36]	Smoking	Low risk	High risk	Unclear	Unclear	High risk	Low risk	Low risk
Lambert et al., 2011, Singapore [30]	Smoking	Low risk	Low risk	Low risk	Low risk	Low risk	Low risk	Low risk
Fritz et al., 2013, USA [32]	Smoking	Low risk	Low risk	High risk	Low risk	High risk	Low risk	Low risk
Zhang et al., 2013, Australia [37]	Smoking	Low risk	Low risk	Low risk	Low risk	High risk	Low risk	Low risk
Baccetti et al., 2015, Italy [38]	Smoking	Low risk	High risk	Unclear	Unclear	High risk	Low risk	High risk
Macklin et al., 2006, USA [39]	Hypertension	Low risk	low risk	Low risk	Unclear	Low risk	Low risk	High risk
Flachskampf et al., 2007, Germany [48]	Hypertension	Low risk	Low risk	Low risk	Unclear	Low risk	Low risk	Unclear
Yin et al., 2007, Korea [44]	Hypertension	Low risk	High risk	Low risk	Low risk	High risk	Low risk	Low risk
Zhang et al., 2008, USA [41]	Hypertension	Low risk	High risk	Low risk	Unclear	Low risk	Low risk	Unclear
Zhang et al., 2009, USA [42]	Hypertension	Low risk	High risk	High risk	Low risk	Low risk	Low risk	High risk
Kim et al., 2012, Korea [40]	Hypertension	Low risk	Low risk	Low risk	Low risk	High risk	Low risk	Unclear
Chen et al., 2013, China [46]	Hypertension	Unclear	Unclear	High risk	Unclear	Unclear	High risk	Unclear
Sriloy et al., 2015, India [49]	Hypertension	Unclear	Low risk	High risk	Unclear	High risk	High risk	High risk
Li et al., 2015, USA [43]	Hypertension	Low risk	High risk	Low risk	Low risk	Low risk	Low risk	Low risk
Liu et al., 2015, Korea [45]	Hypertension	Low risk	Low risk	High risk	Low risk	Low risk	Low risk	High risk
Lin et al., 2016, Taiwan [50]	Hypertension	Low risk	Low risk	Unclear	Unclear	Low risk	Low risk	High risk
Zhan et al., 2016, China [47]	Hypertension	Low risk	High risk	High risk	Unclear	Low risk	Low risk	Unclear
Richards & Marley, 1998, Australia [51]	Obesity	Low risk	High risk	High risk	Unclear	High risk	Low risk	Low risk
Mazzoni et al., 1999, Italy [52]	Obesity	Unclear	Unclear	High risk	Low risk	High risk	Low risk	Low risk
Wei & Liu, 2004, China [53]	Obesity	Unclear	Unclear	High risk	Unclear	Low risk	Unclear	Unclear
Hsu et al., 2005, Taiwan [54]	Obesity	Low risk	High risk	High risk	Unclear	Low risk	Low risk	Low risk
Elder et al., 2007, USA [55]	Obesity	Unclear	Unclear	High risk	Low risk	High risk	High risk	Low risk
Hsieh, 2007, Taiwan [56]	Obesity	Unclear	Unclear	Unclear	Unclear	High risk	Low risk	Unclear
Yeh & Yeh, 2008, Taiwan [57]	Obesity	Low risk	High risk	High risk	High risk	Low risk	Low risk	High risk
Nourshahi et al., 2009, Iran [58]	Obesity	Unclear	Unclear	High risk	Unclear	Low risk	Low risk	Unclear
Hsu et al., 2009, Taiwan [59]	Obesity	Low risk	High risk	Low risk	Unclear	High risk	Low risk	Low risk
Hsieh, 2010, Taiwan [60,61,65]	Obesity	Unclear	Unclear	Unclear	Unclear	High risk	High risk	High risk
Rerksuppaphol & Rerksuppaphol, 2011, Thailand [62]	Obesity	Low risk	High risk	High risk	High risk	Low risk	Low risk	Low risk
Rerksuppaphol, 2012, Thailand [63]	Obesity	Low risk	High risk	High risk	Unclear	High risk	Low risk	High risk
Lien et al., 2012, Taiwan [64]	Obesity	Low risk	High risk	Low risk	Unclear	High risk	Low risk	Low risk
Darbandi et al., 2012, Iran [66]	Obesity	Low risk	High risk	Unclear	Unclear	Low risk	Low risk	Low risk
Abdi et al., 2012, Iran [67]	Obesity	Unclear	Unclear	Unclear	Unclear	High risk	Low risk	Low risk
He et al., 2012, China [68]	Obesity	Unclear	Unclear	High risk	Unclear	Low risk	Low risk	High risk
Guo et al., 2014, China [69]	Obesity	Unclear	Unclear	High risk	Unclear	Low risk	Low risk	High risk
Wu et al., 2014, China [70]	Obesity	Low risk	High risk	Low risk	Unclear	Low risk	Low risk	High risk
Kim et al., 2014, Korea [71]	Obesity	Low risk	High risk	High risk	Unclear	High risk	Low risk	Low risk
Yeo et al., 2014, Korea [72]	Obesity	Low risk	Low risk	High risk	Unclear	High risk	Low risk	High risk
Schukro et al., 2014, Austria [73]	Obesity	Low risk	High risk	High risk	Unclear	High risk	Low risk	Low risk
Darbandi et al., 2014, Iran [74]	Obesity	Low risk	High risk	High risk	High risk	Low risk	Low risk	Low risk
Yeh et al., 2015, Taiwan [75]	Obesity	Low risk	Low risk	low risk	Unclear	High risk	Low risk	High risk
He et al., 2015, China [76]	Obesity	Low risk	High risk	High risk	Unclear	Low risk	Low risk	High risk
Jiao et al., 2015, China [77]	Obesity	Low risk	High risk	High risk	Unclear	Low risk	Unclear	Unclear

^a^Blinding of participants and personnel: We only focus upon the blinding of participants as blinding the acupuncturists in acupuncture treatments is impossible due to methodological reasons.

## Results

**Fig 1:**
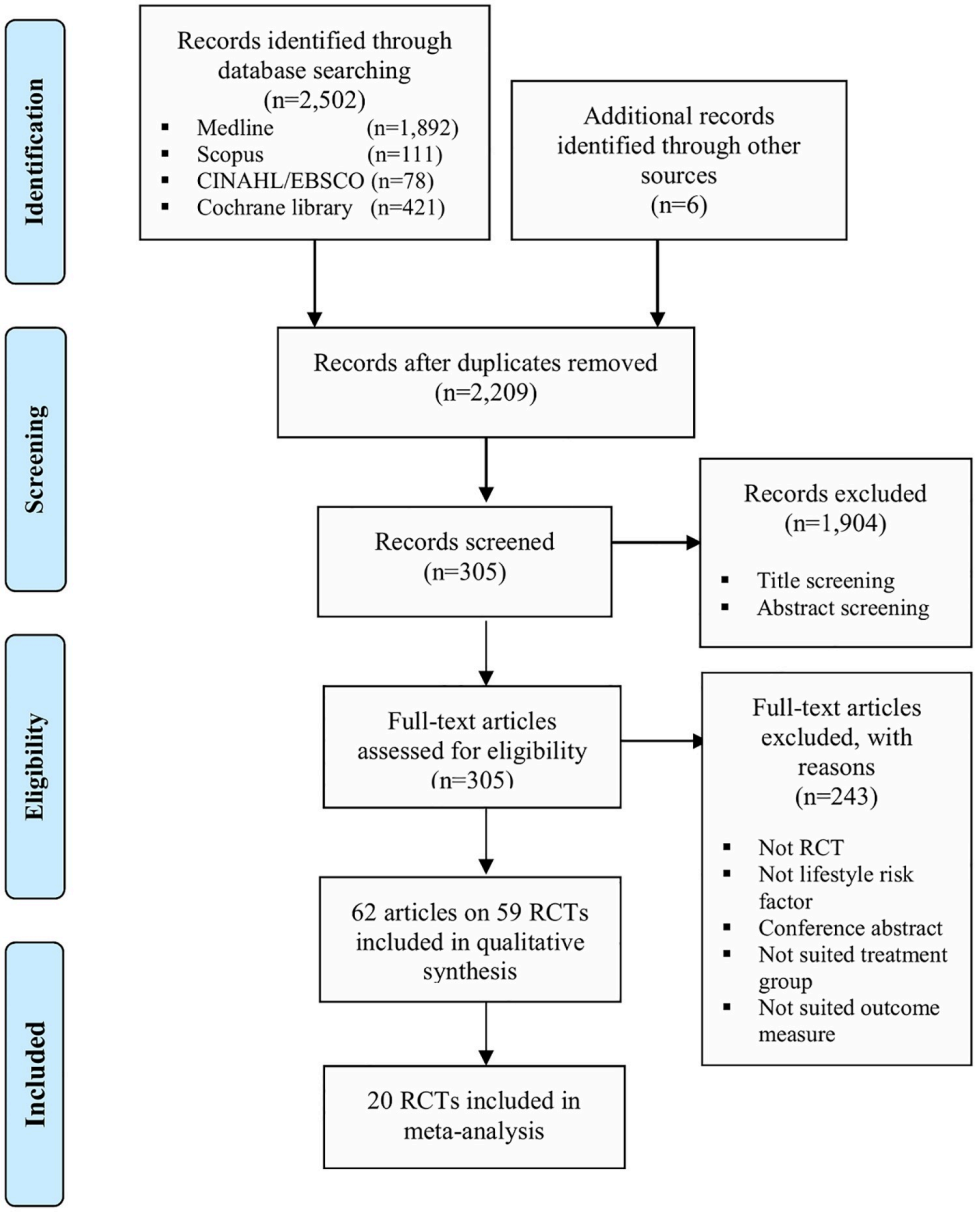
PRISMA flowchart of literature search and study selection.

The key database searches identified 2,502 records with another six records from Google Scholar search, of which 299 duplicates were removed. After screening, the full texts of 305 papers were reviewed, of which a total of 62 full-text articles (reporting on 59 RCTs) were considered eligible and included in this systematic review. The PRISMA flowchart of literature search and article selection details has been shown in Fig 1.

There were 59 RCTs (5,650 participants) regarding the use of acupuncture interventions in treating lifestyle risk factors for stroke, of which 7 RCTs for alcohol-dependence (845 participants), 15 RCTs for smoking-dependence (1,960 participants), 12 RCTs for hypertension (927 participants), and 25 RCTs for obesity (1,918 participants). No publication reported on a trial examining the efficacy of acupuncture for the lifestyle risk factor for stroke of high cholesterol or physical inactivity as a primary outcome.

### Alcohol-dependence

Seven RCTs [16–22] focused on acupuncture treatments for alcohol-dependence using outcomes of alcohol craving (four RCTs), alcohol withdrawal symptoms (four RCTs), and drinking days (one RCT). Table 2 shows details of such RCTs’ characteristics and safety-related information. Most of the included studies defined alcohol-dependence according to the 3^rd^ version (revised)/4^th^ version of the Diagnostic and Statistical Manual of Mental Disorders (DSM) or the 10^th^ version of the International Statistical Classification of Diseases and Related Health Problems (ICD) [16–21]. The sample size of RCTs focusing on alcohol-dependence ranged from 20 to 503 participants with only two studies recruiting more than 100 participants.

Psychiatrists/nurses [17,20], acupuncturists [18,22], and oriental medical doctors [16] were reported as administering the acupuncture interventions. The modes of acupuncture delivered within the interventions included both specific and nonspecific/symptom-based auricular acupuncture (five studies), body acupuncture (one study), and combined auricular and body acupuncture (one study). Acupuncture treatment sessions ranged from 30-minutes to 45-minutes. Only one RCT employed needle stimulation technique for the acupuncture treatment of alcohol-dependence [17].

*Non-significant differences* between acupuncture and control groups for alcohol craving were reported in three RCTs [16,17,20], alcohol withdrawal symptoms in two RCTs [17,18], and drinking days in one RCT [20]. Statistically significant *within-intervention group effects* were reported for alcohol craving with specific auricular electroacupuncture [21] and alcohol withdrawal symptoms with combined use of auricular and body acupuncture [19], while statistically significant *between-group effects* were reported for alcohol withdrawal symptoms with symptom-based auricular acupuncture (VS specific auricular acupuncture) [22].

Risk of bias assessment indicated that three RCTs did not report information on random sequence generation, four RCTs failed to apply blinding to participants and personnel, one did not report adequate blinding of outcome assessors, and three failed to report complete outcome data (Table 4). Due to the great heterogeneity regarding intervention details and outcomes applied in the RCTs focusing on alcohol-dependence, no meta-analysis could be conducted.

### Smoking-dependence

Fifteen RCTs [23–38] focused on acupuncture treatments for smoking-dependence using outcomes of daily cigarette consumption (eight RCTs), smoking cessation rate (eight RCTs), smoking withdrawal symptoms (six RCTs), desire to smoke (two RCTs), cotinine concentrations (one RCT), and craving (one RCT). The details of such RCTs’ characteristics and safety-related information have been presented in Table 2. The majority of these RCTs defined smoking-dependence according to the number of cigarettes daily and/or smoking period [23–30,32–35,37–38]. The sample size of the RCTs ranged from 29 to 477 participants, with six RCTs recruiting more than 100 participants.

Acupuncturists were reported to administer the acupuncture intervention in seven RCTs [23,24,26,31–33,37], while physicians and researchers were reported to administer the acupuncture intervention in two RCTs [25,38] and one RCT [28], respectively. The modes of acupuncture delievered within the RCTs focusing on smoking-dependence included auricular acupuncture (four RCTs), auricular acupressure (three RCTs), body acupuncture (one RCT), TEAS (two RCTs), combined auricular acupuncture and auricular acupressure (two RCTs), combined auricular acupuncture, body acupuncture, and education (one RCT), combined auricular acupressure, body acupuncture, and psychological support (one RCT), and combined auricular acupuncture, body acupuncture, and auricular acupressure (one RCT). A total of 11 RCTs included acupuncture treatment follow-ups [24–29,31,33,36–38] and most ranged between 3 months to 9 months after the treatment. All electroacupuncture RCTs were conducted over 20-minutes (per session) with different stimulation frequency [23–26,32,34].

Study results reported statistically significant *within-intervention group effects* for (a) daily cigarette consumption with combined body electroacupuncture, auricular acupuncture and auricular acupressure [23,24], auricular acupuncture [33], combined auricular electroacupuncture and acupressure [34], auricular acupressure [36], (b) desire to smoke with combined body electroacupuncture, auricular acupuncture and auricular acupressure [23,24], and (c) smoking withdrawal symptoms with auricular acupuncture [33]. Statistically significant *between-group effects* were reported for (a) smoking cessation rate with combined body electroacupuncture, auricular acupuncture and auricular acupressure (VS non-specific acupuncture) [23,24], combined auricular electroacupuncture and acupressure (VS sham acupuncture) [25], combined auricular acupuncture, body acupuncture, and education (VS sham acupuncture plus education) [31], (b) daily cigarette consumption with combined body electroacupuncture, auricular acupuncture and auricular acupressure [23,24], combined auricular acupuncture, body acupuncture, and education [31], (c) desire to smoke with combined body electroacupuncture, auricular acupuncture and auricular acupressure [23,24], TEAS (VS sham TEAS) [30], and (d) smoking withdrawal symptoms with body acupuncture (VS non-specific acupuncture) [35].

Compared to sham acupuncture, meta-analyses demonstrated individuals receiving auricular acupressure for smoking-dependence reported lower numbers of consumed cigarettes per day (two RCTs, MD = -2.75 cigarettes/day; 95%CI: -5.33, -0.17; p = 0.04; heterogeneity: I^2^ = 0%; Chi^2^ = 0.45; p = 0.50). However, none of the effect of these two RCTs was robust against selection bias and performance/detection bias. Meta-analysis did not show evidence for post-intervention effect of acupuncture interventions on smoking withdrawal symptoms compared to sham acupuncture (three RCTs, SMD = -0.95; 95%CI: -2.17, 0.26; p = 0.12). In addition, no evidence from meta-analysis has been found with regards to post-intervention effect on smoking cessation rate compared to sham controls, including acupuncture (three RCTs, RR = 1.11; 95% CI: 0.85, 1.46; p = 0.44), auricular acupressure (two RCTs, RR = 0.39; 95% CI: 0.08, 1.96; p = 0.26), and acupuncture plus auricular acupressure (two RCTs, RR = 2.51; 95% CI: 0.26, 24.24; p = 0.43). There was also no evidence for long-term effect on smoking cessation rate, including acupuncture (two RCTs, RR = 1.13; 95% CI: 0.40, 3.21; p = 0.82), auricular acupressure (two RCTs, RR = 2.43; 95% CI: 0.40, 14.66; p = 0.33), and acupuncture plus auricular acupressure (two RCTs, RR = 1.97; 95% CI: 0.67, 5.80; p = 0.22), when compared to sham controls (Table 3). Risk of bias assessment indicated 13 RCTs applied random sequence generation while nine RCTs did not allocate concealment appropriately. Seven RCTs failed to report information on blinding of outcome assessment. Ten RCTs did not provide complete outcome data (Table 4).

### Hypertension

Twelve RCTs [39–50] focused on acupuncture treatments for hypertension using outcomes of both SBP and DBP (12 RCTs), nighttime SBP and DBP (one RCT), daytime SBP and DBP (one RCT). See Table 2 for details of these RCTs’ characteristics and safety-related information. Most of these RCTs defined hypertension according to the *[varied]* upper and lower cut-off points of SBP and DBP levels with/without antihypertensive medication(s). The sample size of these RCTs ranged from 30 to 160 participants, and three of these studies recruited more than 100 participants.

Acupuncturists [39,46,48], physicians [44], Korean medicine practitioners [40], and naturopaths [49] administered acupuncture for hypertension. The modes of acupuncture delivered within the interventions included body acupuncture (eight RCTs), body acupressure (one RCT), combined body and auricular acupuncture (two RCTs), combined body acupuncture and music treatment (one RCT), and combined body acupuncture and exercise (one RCT). Four RCTs followed the effects of acupuncture interventions up to 12 months after treatment [39,43,45,48]. Seven RCTs using needle acupuncture employed stimulation techniques [39,40,44–47,49].

Both statistically significant *within-intervention group* and *between-group effects* were reported in five RCTs for (a) SBP as well as DBP levels with body acupuncture (VS non-specific acupuncture) [48], combined body acupuncture and exercise (VS sham acupuncture plus exercise) [44], combined laser body acupuncture with/without music treatment (VS starch tablets) [47], body acupressure (VS sham acupuncture) [50], (b) nighttime DBP level with body acupuncture (VS sham acupuncture) [40]. In addition, study results reported statistically significant *within-intervention group effects* for (a) SBP as well as DBP levels with laser acupuncture [41], (b) SBP level with body electroacupuncture [42], (c) DBP level with combined body and auricular acupuncture [49], and statistically significant *between-group effect* for SBP level with body electroacupuncture (VS sham acupuncture) [43].

Meta-analyses did not show evidence for neither post-intervention nor long-term effect of acupuncture interventions on SBP control (two RCTs on acupuncture, MD = -0.54 mmHg; 95%CI: -10.69, 9.60; p = 0.92) and DBP control (two RCTs on acupuncture, MD = -1.38 mmHg; 95%CI: -4.06, 1.31; p = 0.32) compared to sham acupuncture (Table 3). Risk of bias assessment indicated only six hypertension-focused RCTs blinded participants and personnel appropriately and seven RCTs did not report information on blinding of outcome assessment (Table 4).

### Obesity

A total of 25 RCTs [51–77] focused on acupuncture treatments for obesity using outcomes of BMI (19 RCTs), BW (including weight loss) (18 RCTs), WC (11 RCTs), hip circumstance (four RCTs), eating suppression (two RCTs), waist-to-hip ratio (two RCTs), and fat mass (two RCTs). See Table 2 for details of the characteristics and safety-related information of these studies. Most of these RCTs defined obesity according to participants’ BMI with/without WC [52–57,59–77]. The sample size of these 25 RCTs ranged from 27 to 196 participants, and three of these studies recruited more than 100 participants.

Among the 11 obesity-focused RCTs that specified the personnel who administered acupuncture, acupuncturists were chosen in nine RCTs [52,55,58,59,62–64,66,67]. The modes of acupuncture delivered within the interventions included auricular acupressure (six RCTs), auricular acupuncture (four RCTs), body acupuncture (four RCTs), Tapas acupressure or TEAS (two RCTs), combined auricular acupuncture and auricular acupressure (one RCT), combined auricular and body acupuncture with/without other intervention(s) (ie. moxibustion, exercise, diet) (three RCTs), auricular acupressure with TEAS or exercise (two RCTs), and body acupuncture with exercise, diet, or massage (three RCTs). Three obesity-focused RCTs followed the effect of acupuncture interventions, from 10-weeks to 12-months after the treatment [66,67,73]. All the electroacupuncture/TEAS studies focusing on BW employed different stimulation frequency with varied treatment durations [54,58,62,63,67,69,70,73,75,77].

Study results reported statistically significant *within-intervention group effects* for all BW, BMI, and WC with auricular acupressure (BW [60,61,64–66,71]; BMI [56,60,61,64–66,71]; WC [60,61,64,65]), combined auricular acupressure and TEAS [63], combined auricular acupressure and exercise [68], and body acupuncture [67,70]. Additionally, study results reported statistically significant *within-intervention group effects* for both BW and BMI with TEAS [62] and combined body acupuncture and massage [76]. Statistically significant *between-group effects* were reported for all BW, BMI, and WC with auricular acupressure (BW [61,61,65,71]; BMI [71]; WC [60,61,65]), auricular acupuncture (BW [51,72,73]; BMI [72,73]; WC [54,74]), and body acupuncture (BW [54]; BMI [54,74]; WC [54,74]). Combined body acupuncture and auricular acupuncture with/without exercise and diet has also shown statistically significant *between-group effects* for BW [53] and BMI [58], respectively.

Relative to sham acupuncture, meta-analyses only found those receiving acupuncture interventions for obesity reported lower waist circumference (five RCTs, MD = -2.79 cm; 95% CI: -4.13, -1.46; p<0.001; heterogeneity: I^2^ = 0%; Chi^2^ = 1.61; p = 0.81). However, after excluding RCTs with other than low risks of selection and performance/detection bias, none of the effect remained statistically significant. In comparison with no treatment intervention, meta-analyses did not show evidence for post-intervention effect of acupuncture interventions on BW (two RCTs on acupuncture, MD = -1.12 kg; 95%CI: -5.51, 3.27; p = 0.62; two RCTs on auricular acupressure, MD = -2.87 Kg; 95%CI: -6.47, 0.74; p = 0.12). Meta-analyses also did not show evidence for post-intervention effect of auricular acupressure interventions on BMI (two RCTs, MD = -0.41 kg/m^2^; 95%CI: -1.56, 0.73; p = 0.48) compared to no treatment (Table 3). Risk of bias assessment was unclear in numerous obesity-focused RCTs due to a lack of detail in the publications. Specifically, nine RCTs did not report random sequence generation and allocation concealment information. Twelve RCTs failed to report complete outcome data. Fifteen RCTs did not blind participants and personnel and 20 RCTs did not provide information on blinding of outcome assessment (Table 4).

## Discussion

This article reports the first systematic review of the effect of acupuncture interventions for lifestyle risk factors for stroke. A number of acupuncture techniques have been used for the management of these lifestyle risk factors and have yielded limited improvements in outcomes. No analysis can be conducted on RCTs focusing on alcohol-dependence and no evidence of the effect of acupuncture treatments on high blood pressure was shown based on meta-analysis. The meta-analysis showed individuals receiving auricular acupressure reported better outcomes in daily cigarette consumption than sham acupressure. Furthermore, acupuncture users have reported better outcomes in reducing waist circumference compared to sham acupuncture. No serious side effects occurred when using acupuncture on these four lifestyle risk factors. However, approximately half of the RCTs focusing on hypertension and obesity did not report safety information of acupuncture users. As such, acupuncture appears to be a relative safe treatment for the management of lifestyle risk factors for stroke.

Some evidence of the benefits of acupuncture and/or auricular acupressure was revealed for RCTs of lifestyle risk factors for stroke—smoking-dependence and obesity—in our review. However, a total of eight and 14 types of acupuncture-related interventions have been examined in RCTs focusing on smoking-dependence and obesity, respectively. The findings reported here highlighted the gaps in the evidence of clinical acupuncture use in the specific field of lifestyle risk factors for stroke and generally. Consistent with findings of prior systematic reviews [9,78], acupuncture involves a range of techniques. Both acupuncture-associated clinical trials and observational studies are required to determine methodology issues such as the use of acupuncture only, acupressure only, or the combination of acupuncture and acupressure, and the further choices of acupuncture like needle acupuncture, electroacupuncture and laser acupuncture. Therefore, future high-quality research is warranted to confirm our preliminary findings and provide robust effect estimates of acupuncture interventions for lifestyle risk factors for stroke.

In our review, approximately half of the RCTs focusing on smoking-dependence and obesity employed auricular acupressure alone or in combination with other acupuncture intervention(s). Acupressure is considered more practical (ease of application by patients themselves) with low cost, compared to other acupuncture treatments [79]. However, no consistent and convincing evidence has been found in this review on whether acupressure is effective for the management of overall lifestyle risk factors for stroke. As a result, there is insufficient evidence to conclude that the use of acupressure could improve the lifestyle risk factors for stroke and more studies are required.

Sham acupuncture is the most frequently employed comparison for acupuncture treatments in general [80] and among people with lifestyle risk factors for stroke which has been shown in our review. Although meta-analysis presented here reported statistically significant benefits of real acupuncture interventions regarding the management of the lifestyle risk factors of smoking-dependence and obesity than sham interventions, none of the effects of the RCTs included in the analyses was robust against potential selection, performance, and detection bias. In addition to the identified design challenges of acupuncture-associated RCTs regarding the choice of control group with the fact that sham acupuncture may also trigger physiological effect [81], future acupuncture-associated RCTs should avoid high risk of bias from lack of allocation concealment and missing outcome data, persuade original investigators to provide sufficient information on blinding of outcome ascertainment and if necessary, choose an appropriate comparable control intervention for clinical acupuncture research.

Some limitations of our systematic review are worth noting. The acupuncture interventions varied greatly across the RCTs of each lifestyle risk factor for stroke included in this review in terms of inclusion criteria of participants, acupuncture forms, acupoint selection, manipulation methods, and frequency/duration of the treatments. Also, this systematic review was restricted to RCTs published in English-language peer-reviewed journals. Furthermore, a proportion of included studies were not registered before they were published, we therefore cannot rule out the possibility of reporting or publication bias. The findings in this systematic review regarding the effect of acupuncture for lifestyle risk factors for stroke should be interpreted with caution. However, compared to previous Cochrane and systematic reviews [9,12,13,82], based on the risk of bias evaluation (Table 4), the methodological quality of RCTs on acupuncture treatments identified in our review has improved over recent years, including regards to random sequence generation application, the reporting of acupuncture treatments, and use of long-term follow-ups.

## Conclusion

This review shows no convincing evidence regarding the effect of acupuncture, acupressure, laser acupuncture or their combination use for lifestyle risk factors for stroke. However, the translation of findings of this systematic review may contribute to the evidence-base of potential clinical practice guideline recommendations for stroke prevention.

## Supporting information

S1 File PRISMA checklist. (DOC)

S2 File PROSPERO protocol registration. (PDF)
