# Supplementary material for: Efficacy of acupuncture for lifestyle risk factors for stroke: A systematic review
Source: PLoS One. 2018 Oct 26;13(10):e0206288. doi: 10.1371/journal.pone.0206288 (PMC6203376; doi:10.1371/journal.pone.0206288)
Supplement: S2 File — (PDF) [file pone.0206288.s002.pdf]

Efficacy of acupuncture/moxibustion and acupressure for stroke prevention: a systematic review

David Sibbritt, Jon Adams, Caleb Ferguson, Jane Frawley, Romy Lauche, Wenbo Peng

Citation

David Sibbritt, Jon Adams, Caleb Ferguson, Jane Frawley, Romy Lauche, Wenbo Peng. Efficacy of acupuncture/moxibustion and acupressure for stroke prevention: a systematic review. PROSPERO 2017:CRD42017060490 Available from
http://www.crd.york.ac.uk/PROSPERO_REBRANDING/display_record.asp?ID=CRD42017060490

Review question(s)

To identify and summarise the evidence of acupuncture/moxibustion and acupressure interventions for the prevention of stroke for those people at greatest risk of stroke.

Searches

Sources: MEDLINE, CINAHL/EBSCO, Scopus, Google Scholar, and Cochrane Database of Systematic Reviews.

Search terms relevant to ‘acupuncture’, 'moxibustion', and 'acupressure' as well as terms regarding a range of ‘stroke risk factors’ including high blood pressure, high cholesterol, irregular pulse, transient ischaemic attack, diabetes, and obesity.

Restrictions: peer-reviewed English language journal articles published from 1996 to 2016.

Types of study to be included

Randomised controlled trials and cross-over studies.

Condition or domain being studied

Stroke prevention;

Hypertension (including mild level hypertension);

Diabetes (including impaired glucose tolerance);

Hypercholesterolemia;

Overweight/Obesity;

Atrial fibrillation;

Transient ischaemic attack.

Participants/ population

General population for stroke prevention and those with increased stroke risk due to hypertension, diabetes, hypercholesterolemia, overweight or obesity, atrial fibrillation, and/or transient ischaemic attack.

Intervention(s), exposure(s)

Inclusion criteria: any acupuncture, moxibustion, acupressure interventions. There is no limitation on the forms of acupuncture, moxibustion, and acupressure (e.g. auricular acupuncture, electroacupuncture, laser acupuncture, acupoint stimulation) and the frequency and duration of the intervention.

Comparator(s)/ control

Any comparator(s)/control.

Outcome(s)

Primary outcomes

1. Stroke incidence rate for stroke prevention trials.

2. Blood pressure for hypertension trials.

3. Triglycerides and total/LDL/HDL cholesterol for hypercholesterolemia trials.

4. Weight, body mass index, waist/hip circumference, and body fat for overweight/obesity trials.

5. Insulin, fasting or postprandial blood glucose, HbA1c and the HOMA index for diabetes trials.

6. Irregular pulse for atrial fibrillation trials.

7. Transient ischaemic attack trials.

8. Safety for all trials.

Secondary outcomes

None.

Data extraction, (selection and coding)

Titles and abstracts of all citations via initial search will be imported to Endnote and duplicates will be removed. Two authors will independently screen all these information to identify articles meeting the inclusion and exclusion criteria. Abstracts mentioning the key words will be selected for full-text review. All unclear studies will be assessed by a third author. In addition, one author will screen the reference lists of papers included in this systematic review paper.

Two authors will independently extract data into a pre-determined data extraction form. Differences in data extraction and interpretation will be resolved through discussion amongst all authors. The form will include information on country/year, sample characteristics, intervention, outcome measure(s), results, limitations, and side effects.

Risk of bias (quality) assessment

Two authors will independently assess risk of bias using the Cochrane Risk of Bias Tool for selection bias (random sequence generation and allocation concealment), performance bias (blinding of participants and personnel), detection bias (blinding of outcome assessment), attrition bias (incomplete outcome data), reporting bias (selective outcome reporting), and other sources of bias. Disagreements will be resolved through discussion with a third author.

The risk of bias assessment will be used to conduct sensitivity analyses to identify the level (high/low/unclear) of bias of each included study.

Strategy for data synthesis

The narrative (descriptive) synthesis will be undertaken on eligible studies.

Analysis of subgroups or subsets

None is planned.

Dissemination plans

Publication in a peer-reviewed journal.

Contact details for further information

Professor Sibbritt

Level 8, Building 10, University of Technology Sydney, 235 Jones St, Ultimo, NSW, Australia 2007

David.Sibbritt@uts.edu.au

Organisational affiliation of the review
None.

Review team
Professor David Sibbritt, University of Technology Sydney
Professor Jon Adams, University of Technology Sydney
Dr Caleb Ferguson, University of Technology Sydney
Dr Jane Frawley, University of Technology Sydney
Dr Romy Lauche, University of Technology Sydney
Dr Wenbo Peng, University of Technology Sydney

Anticipated or actual start date
01 February 2017

Anticipated completion date
30 June 2017

Funding sources/sponsors
This review was funded by a grant from the Nancy & Vic Allen Stroke Prevention Fund.

Conflicts of interest
None known

Language
English

Country
Australia

Subject index terms status
Subject indexing assigned by CRD

Subject index terms
Acupressure; Acupuncture; Acupuncture Therapy; Humans; Moxibustion; Stroke

Stage of review
Ongoing

Date of registration in PROSPERO
29 March 2017

Date of publication of this revision
29 March 2017

Stage of review at time of this submission	Started	Completed
Preliminary searches	Yes	Yes
Piloting of the study selection process	Yes	Yes
Formal screening of search results against eligibility criteria	Yes	Yes
Data extraction	Yes	No
Risk of bias (quality) assessment	Yes	No
Data analysis	Yes	No

PROSPERO
International prospective register of systematic reviews
The information in this record has been provided by the named contact for this review. CRD has accepted this information in good faith and registered the review in PROSPERO. CRD bears no responsibility or liability for the content of this registration record, any associated files or external websites.
